# Clinical characteristics and prognostic factors of pneumonia in patients with and without rheumatoid arthritis

**DOI:** 10.1371/journal.pone.0201799

**Published:** 2018-08-03

**Authors:** Aya Wakabayashi, Takashi Ishiguro, Yotaro Takaku, Yosuke Miyahara, Naho Kagiyama, Noboru Takayanagi

**Affiliations:** Department of Respiratory Medicine, Saitama Cardiovascular and Respiratory Center, Kumagaya, Saitama, Japan; Auburn University College of Veterinary Medicine, UNITED STATES

## Abstract

**Background:**

To elucidate the characteristics of pneumonia in rheumatoid arthritis (RA) patients and to assess whether pneumonia in RA patients differs from that in non-RA patients.

**Methods:**

We retrospectively divided pneumonia patients into two groups, those with RA and those without RA, and compared the two groups. We evaluated the risk factors for mortality with univariate and multivariate logistic regression analysis.

**Results:**

Among 1549 patients, 71 had RA. The RA patients with pneumonia were 71.0±8.9 years old, 54.9% were female, 40.9% had a smoking history, and 71.8% had underlying respiratory disease. Female sex, non-smoker, and respiratory comorbidities were statistically more frequent in the RA patients than non-RA patients. The most frequent causative microbial agents of pneumonia in the RA patients were *Streptococcus pneumoniae*, *Pseudomonas aeruginosa*, *Haemophilus influenzae*, *Mycoplasma pneumoniae*, and influenza virus, whereas those of pneumonia in non-RA patients were *S*. *pneumoniae*, influenza virus, *M*. *pneumoniae*, *Legionella* spp., *P*. *aeruginosa*, *H*. *influenzae*, and *Moraxella catarrhalis*. Polymicrobial infection were identified as etiologies more frequently in the RA patients than non-RA patients. Although the severity of pneumonia did not differ between the two groups, mortality was statistically higher in the RA patients than non-RA patients. Multivariate analysis showed RA to be an independent risk factor for mortality.

**Conclusions:**

*P*. *aeruginosa*, *H*. *influenzae*, *M*. *catarrhalis*, and polymicrobial infection were statistically more frequent etiologies of pneumonia in the RA patients than non-RA patients. RA itself was found to be an independent risk factor for mortality from pneumonia.

## Introduction

Rheumatoid arthritis (RA) is a chronic systemic inflammatory disease that results in worsening of physical function and extra-articular manifestations. Because of these manifestations, patients with RA have been reported to have a shorter life expectancy than controls [1,2] and are at increased risk of developing infections compared with the general population [3,4]. Furthermore, although many studies have reported favorable results of treatment, since the introduction of a new class of anti-cytokine drugs, problems associated with increased rates of serious infection in RA patients have emerged.

Lower respiratory tract infections are the most frequent infectious events occurring in RA patients [5], and bacterial pneumonia is associated with increased mortality in RA patients [4–6]. A nested case-control study that included 17,172 cases of pneumonia diagnosed between 1996 and 2005 determined that RA was an independent risk factor for community-acquired pneumonia (odds ratio, 1.84; 95% confidence interval [CI], 1.62–2.10) [7]. Recently, a favorable effect of vaccination against influenza and pneumococcus to reduce the prevalence of hospitalized cases of pneumonia in RA patients has been reported [8,9].

Pneumonia may be one of the most important infectious complications of RA patients, but there have been no reports investigating the etiology, severity, and mortality of pneumonia in these patients. Therefore, the objectives of the present study were to clarify the characteristics, etiology, severity, outcome, and prognostic factors of pneumonia in RA patients in comparison with non- RA patients.

## Materials and methods

### Patients and study design

We performed a retrospective study of patients treated with pneumonia at our institution in Saitama, Japan, between January 2002 and December 2011. Characteristics, severity, outcome, and etiology of pneumonia in the RA patients were compared with those in the non-RA patients also treated in our institution during the same period. The following variables were assessed as possible risk factors for mortality from pneumonia: age (≥65 years), male sex, smoking habit, presence of comorbid illness, respiratory comorbidity, RA patient, severe pneumonia, and healthcare-associated pneumonia (HCAP), which we previously reported as risk factors for mortality from CAP [10]. RA was diagnosed by rheumatologists certified by the Japan College of Rheumatology according to established classification criteria [11]. The study protocol was approved by the Ethics Committee of Saitama Cardiovascular and Respiratory Center (Approval no. 2012041) and waived patient consent because this was a retrospective study and anonymity was secured. The study was conducted in compliance with the Declaration of Helsinki.

### Definition of pneumonia

Pneumonia was diagnosed on the basis of symptoms suggestive of lower respiratory tract infection and the development of new infiltrations on chest X-ray. HCAP was defined when the criteria of the American Thoracic Society (ATS)/Infectious Diseases Society of America (IDSA) guidelines [12] were satisfied. Severe pneumonia was defined when at least one major criterion or three minor criteria of the IDSA/ATS guidelines [13] were present. Patients with acquired immunodeficiency syndrome, tuberculosis, non-resected lung cancer, or a confirmed alternative diagnosis until the end of follow-up were excluded from the study.

### Microbiological identification

Diagnosis of causative microorganisms was based on the results of semiquantitative cultures of respiratory samples or blood, paired sera, urinary antigen tests for *Streptococcus pneumoniae* and *Legionella pneumophila*, and nasopharyngeal swabs for influenza virus as reported previously [10]. The antibiotic regimen was chosen by the attending physician in accordance with the recommendations of the ATS/IDSA guidelines [12].

### Statistical analysis

Results are presented as the frequency and percentage or mean ± SD, unless otherwise indicated. Risk factors for mortality from pneumonia in RA patients were evaluated with univariate and multivariate logistic regression analysis. Variables showing significance by univariate analysis were included in the multivariate logistic regression analysis with backward elimination method. The 95% CI for all comparisons is also reported. In all instances, a two-tailed *P* value of ≤ 0.05 was considered to indicate statistical significance. All statistical analyses were performed with Statistical Analysis System software (SAS version 9.1.3; SAS Institute, Inc., Cary, NC).

## Results

### Characteristics of pneumonia patients with and without RA

Overall, 71 patients with RA and 1478 patients without RA were investigated. Characteristics of the RA patients with pneumonia are shown in Table 1. Among these patients with RA, the age at diagnosis was 71.0 ± 8.9 years, 39 (54.9%) patients were female, and 29 (40.9%) patients had a smoking history. Underlying respiratory diseases were identified in 51 (71.8%) patients, and systemic comorbidities were identified in 33 (46.5%) patients. The ratio of females (54.9% vs 30.5%, p < 0.001) and the frequency of underlying respiratory disease (71.8% vs 52.2%, p = 0.001) were significantly higher in the RA patients, but the presence of a smoking history (40.9% vs 62.1%, p < 0.001) was significantly higher in the non-RA patients.

**Table 1:** Characteristics of the patients with and without RA.

Characteristic	RA (*n* = 71)	Non-RA (*n* = 1478)	p value
Age (years)	71.0 ± 8.9^a^	66.9 ± 17.1	<0.001
≥65 years	53 (74.6)	956 (64.7)	0.097
Female sex	39 (54.9)	451 (30.5)	<0.001
Smokers	29 (40.8)	918 (62.1)	<0.001
HCAP	23 (32.4)	484 (32.7)	1.000
Comorbidity			
None	10 (14.1)	339 (22.9)	0.008
Pulmonary disease	51 (71.8)	772 (52.2)	0.001
COPD	12 (16.9)	324 (21.9)	0.377
Asthma	1 (1.4)	149 (10.1)	0.012
Bronchiectasis	9 (12.7)	91 (6.2)	0.043
Nontuberculous mycobacteriosis	10 (14.1)	65 (4.4)	0.002
Old pulmonary tuberculosis	3 (4.2)	105 (7.1)	0.477
Chronic pulmonary aspergillosis	1 (1.4)	25 (1.7)	1.000
Interstitial pneumonia	21 (29.6)	85 (5.8)	<0.001
Post lung cancer operation	1 (1.4)	57 (3.9)	0.517
Other pulmonary disease^b^	12 (16.9)	56 (3.8)	<0.001
Systemic disease	33 (46.5)	655 (44.3)	0.716
Steroid or immunosuppressant	46 (64.8)	87 (5.9)	<0.001
Severe	7 (9.9)	218 (14.7)	0.303
Mortality	8 (11.3)	74 (5.0)	0.050

RA, rheumatoid arthritis; HCAP, healthcare-associated pneumonia; COPD, chronic obstructive pulmonary disease.

^a^Data are presented as means ± standard deviation or n (%).

^b^“Other pulmonary disease” includes the following incidences of each disease: bronchiolitis (n = 6), chronic eosinophilic pneumonia (n = 2), and others (n = 5) in the RA patients, and pneumoconiosis (n = 13), sarcoidosis (n = 9), chronic empyema (n = 8), pulmonary thromboembolism (n = 6), asbestos pleuritis (n = 4), pulmonary hypertension, alveolar proteinosis (n = 3 each), and others (n = 10) in the non-RA patients. Some patients had more than one comorbidity.

Compared with RA patients without underlying respiratory disease, the mean age of those having respiratory comorbidities was significantly older and the rate of male sex was significantly higher (Table 2). Therapeutic drugs for RA were administered in 57 patients (biologics [*n* = 5], methotrexate [MTX] [*n* = 17], corticosteroid [*n* = 31], and DMARDs [disease-modifying anti-rheumatic drugs] other than MTX [*n* = 27]). Patients with respiratory comorbidities tended to be less frequently treated with MTX or biologics (Table 2). There were no differences in the characteristics of the RA patients between those with severe versus non-severe pneumonia (Table 2). Compared with the RA patients who did not survive, those who did survive tended to be more frequently treated with a steroid or immunosuppressant.

**Table 2:** Characteristics of the patients with RA.

Characteristic	Pulmonary comorbidity			Therapeutic agents for RA			Severity			Non-Survivors		
	Present (*n* = 51)	None (*n* = 20)	p value	Biological agent and/or MTX (*n* = 18)	Others (*n* = 53)	p value	Severe (*n* = 7)	Non-severe (*n* = 64)	p value	Non-survivors (*n* = 8)	Survivors (*n* = 63)	p value
Age (years)	73.0 ± 8.1^a^	66.1 ± 9.1	0.002	68.1 ± 10.0	72.0 ± 8.3	0.100	74.4 ± 8.4	70.7 ± 8.9	0.289	73.9 ± 7.4	70.7 ± 9.0	0.339
Female sex	24 (47.1)	15 (75.0)	0.038	10 (55.6)	29 (54.7)	1.000	2 (28.6)	37 (57.8)	0.231	4 (50.0)	35 (55.6)	1.000
Smokers	24 (47.1)	5 (25.0)	0.112	7 (38.9)	22 (41.5)	1.000	4 (57.1)	25 (39.1)	0.433	2 (25.0)	27 (42.9)	0.458
HCAP	18 (35.3)	5 (25.0)	0.574	5 (27.8)	18 (34.0)	0.774	1 (14.3)	22 (34.4)	0.415	1 (12.5)	22 (34.9)	0.261
Steroid or immunosuppressant	31 (60.8)	15 (75.0)	0.287	18 (100.0)	28 (52.8)	<0.001	2 (28.6)	44 (68.8)	0.088	2 (25.0)	44 (69.8)	0.019
Comorbidity												
None	0 (0.0)	10 (50.0)	<0.001	5 (27.8)	5 (9.4)	0.036	2 (28.6)	8 (12.5)	0.687	0 (0.0)	10 (15.9)	0.254
Pulmonary disease	51 (100.0)	0 (0.0)	<0.001	9 (50.0)	42 (79.2)	0.031	4 (57.1)	47 (73.4)	0.394	8 (100.0)	43 (68.3)	0.095
Systemic disease	23 (45.1)	10 (50.0)	0.794	6 (33.3)	27 (50.9)	0.275	3 (42.9)	30 (46.9)	1.000	5 (62.5)	28 (44.4)	0.459
Severe	4 (7.8)	3 (15.0)	0.394	2 (11.1)	5 (9.4)	1.000	7 (100.0)	0 (0.0)	<0.001	3 (37.5)	4 (6.3)	0.027
Mortality	8 (15.7)	0 (0.0)	0.095	0 (0.0)	8 (15.1)	0.105	3 (42.9)	5 (7.8)	0.027	8 (100.0)	0 (0.0)	<0.001

RA, rheumatoid arthritis; HCAP, healthcare-associated pneumonia.

^a^Data are presented as means ± standard deviation or n (%).

### Microbiological etiology of pneumonia in patients with and without RA

Pathogens were identified in 40 (56.3%) of the RA patients and 824 (55.8%) of the non-RA patients (Table 3). The five most frequently isolated pathogens in the RA patients were *S*. *pneumoniae* (18.3%), *Pseudomonas aeruginosa* (14.1%), *Haemophilus influenzae* (9.9%), *Mycoplasma pneumoniae*, and influenza virus (8.5% each). In contrast, the four most frequently isolated pathogens in the non-RA patients were *S*. *pneumoniae* (23.2%), influenza virus (9.4%), *M*. *pneumoniae* (8.1%), and *Legionella* spp. (4.7%) (Table 3). *P*. *aeruginosa* (14.1% vs 4.5%, p = 0.002), *H*. *influenzae* (9.9% vs 4.2%, p = 0.035), *M*. *catarrhalis* (7.0% vs 0.9%, p = 0.001), and polymicrobial infection (23.9% vs 8.3%, p < 0.001) were identified as etiologies statistically more frequently in the RA patients than in the non-RA patients (Table 3).

**Table 3:** Etiology of pneumonia in the RA and non-RA patients.

Etiology	RA (*n* = 71)		Non-RA (*n* = 1478)		p value
	No.	(%)	No.	(%)	
*Streptococcus pneumoniae*	13	18.3	343	23.2	0.388
Influenza virus	6	8.5	139	9.4	1.000
*Mycoplasma pneumoniae*	6	8.5	119	8.1	0.824
*Legionella* spp.	1	1.4	70	4.7	0.253
*Pseudomonas aeruginosa*	10	14.1	67	4.5	0.002
*Haemophilus influenzae*	7	9.9	62	4.2	0.035
GNEB	4	5.6	48	3.2	0.296
*Chlamydophila pneumoniae*	2	2.8	30	2.0	0.656
*Chlamydophila psittaci*	0	0.0	16	1.1	1.000
*Moraxella catarrhalis*	5	7.0	14	0.9	0.001
*Streptococcus* sp.^a^	0	0.0	12	0.8	1.000
*Stenotrophomonas maltophilia*	1	1.4	1	0.1	0.090
Others^b^	5	7.0	31	2.1	0.022
Polymicrobial infection^c^	17	23.9	123	8.3	<0.001
Unknown	31	43.7	654	44.2	1.000

RA, rheumatoid arthritis; GNEB, Gram-negative enteric bacilli.

^a^*Streptococcus* sp. means other than *S*. *pneumoniae*.

^b^“Others” includes coagulase-negative *Staphylococci* (*n* = 3), and methicillin-susceptible *Staphylococcus aureus*, *Haemophilus parainfluenzae* (*n* = 1 each) in the RA patients, and methicillin-susceptible *S*. *aureus* (*n* = 11), methicillin-resistant *S*. *aureus* (*n* = 5), *Nocardia* sp. (*n* = 3), *H*. *parainfluenzae* (*n* = 2), and coagulase-negative *Staphylococci*, *S*. *haemolyticus*, *Acinetobacter baumannii*, *A*. *calcoaceticus*, Varicella-zoster virus, *H*. *parahaemolyticus*, *Pseudomonas alcaligenes*, *Citrobacter freundii* (*n* = 1 each), and anaerobes (*n* = 7) (*Fusobacterium* sp. [*n* = 2], and *Clostridium perfringens*, *Prevotella* sp., *Gemella morbillorum*, *Peptostreptococcus prevostii*, and *Veillonella* sp. [*n* = 1 each]) in the non-RA patients.

^c^“Polymicrobial infection” includes *S*. *pneumoniae* + *M*. *pneumoniae*, *H*. *influenzae* + influenza virus, *H*. *influenzae* + *M*. *catarrhalis* and *P*. *aeruginosa* + GNEB (*n* = 2 each) and *S*. *pneumoniae* + *H*. *influenzae*, *S*. *pneumoniae* + influenza virus, *H*. *influenzae* + *P*. *aeruginosa*, *M*. *pneumoniae* + methicillin-susceptible *S*. *aureus*, *P*. *aeruginosa* + *H*. *parainfluenzae*, influenza virus + coagulase-negative *Staphylococci*, *S*. *pneumoniae* + *M*. *catarrhalis* + coagulase-negative *Staphylococci*, *S*. *pneumoniae* + influenza virus + *C*. *pneumoniae* and *H*. *influenzae* + *P*. *aeruginosa* + *C*. *pneumoniae* (*n* = 1 each) in the RA patients, and *S*. *pneumoniae* + influenza virus (*n* = 30), *S*. *pneumoniae* + *H*. *influenza* (*n* = 13), *S*. *pneumoniae* + *C*. *pneumoniae* (*n* = 8), *H*. *influenzae* + influenza virus (*n* = 6), *S*. *pneumoniae* + *Legionella* spp. (*n* = 5), *S*. *pneumoniae* + *M*. *pneumoniae*, *M*. *pneumoniae* + influenza virus and *H*. *influenzae* + *P*. *aeruginosa* (*n* = 4 each), *S*. *pneumoniae* + *M*. *catarrhalis* and influenza virus + *Legionella* spp. (*n* = 3 each), *S*. *pneumoniae* + *P*. *aeruginosa*, *S*. *pneumoniae* + methicillin-susceptible *S*. *aureus*, *H*. *influenzae* + *M*. *pneumoniae*, *M*. *pneumoniae* + *P*. *aeruginosa*, *M*. *pneumoniae* + *Legionella* spp., influenza virus + GNEB, *P*. *aeruginosa* + methicillin-resistant *S*. *aureus*, *P*. *aeruginosa* + *Legionella* spp. and *S*. *pneumoniae* + *M*. *catarrhalis* + influenza virus (*n* = 2 each) and *S*. *pneumoniae* + *C*. *psittaci*, *S*. *pneumoniae* + GNEB, *H*. *influenzae* + *M*. *catarrhalis*, *H*. *influenzae* + *C*. *psittaci*, *M*. *pneumoniae* + *C*. *pneumoniae*, *M*. *pneumoniae* + methicillin-susceptible *S*. *aureus*, influenza virus + *P*. *aeruginosa*, influenza virus + *M*. *catarrhalis*, influenza virus + *C*. *pneumoniae*, influenza virus + methicillin-resistant *S*. *aureus*, *C*. *pneumoniae* + *Legionella spp*., *S*. *maltophilia* + methicillin-resistant *S*. *aureus*, *Streptococcus* sp. + GNEB, methicillin-susceptible *S*. *aureus* + *Nocardia* sp., *Prevotella* sp. + *Fusobacterium* sp., *S*. *pneumoniae* + *H*. *influenzae* + influenza virus, *S*. *pneumoniae* + *H*. *influenzae* + methicillin-susceptible *S*. *aureus*, *S*. *pneumoniae* + influenza virus + methicillin-susceptible *S*. *aureus*, *S*. *pneumoniae* + influenza virus + *Legionella* spp., *S*. *pneumoniae* + influenza virus + *C*. *pneumoniae*, *S*. *pneumoniae* + *M*. *catarrhalis* + *C*. *pneumoniae*, *M*. *pneumoniae* + *Streptococcus* sp. + *Fusobacterium* sp., influenza virus + *P*. *aeruginosa* + *A*. *calcoaceticus*, *P*. *aeruginosa* + *P*. *alcaligenes* + *H*. *parahaemolyticus*, and *G*. *morbillorum* + *P*. *prevostii* + *Veillonella* sp. (*n* = 1 each) in the non-RA patients.

The four most frequently isolated pathogens in the RA patients with underlying respiratory diseases were *P*. *aeruginosa* (19.6%), *S*. *pneumoniae* (15.7%), *H*. *influenzae* (13.7%), and *M*. *catarrhalis* (9.8%) (Table 4). All of the patients with *P*. *aeruginosa*, *H*. *influenzae*, and *M*. *catarrhalis* infection had respiratory comorbidities. Pathogens isolated in the non-survivors with RA were *S*. *pneumoniae*, *P*. *aeruginosa* (*n* = 3 each), *M*. *pneumoniae*, *H*. *influenzae*, and *M*. *catarrhalis* (*n* = 1 each) (Table 4). There were no significant differences in etiologies between the RA patients with or without underlying respiratory disease, between the RA patients treated or not-treated with MTX or biologics, between the severe and non-severe RA patients, and between the non-surviving and surviving RA patients.

**Table 4:** Etiology of pneumonia in RA.

Etiology	Pulmonary comorbidity			Therapeutic agents of RA			Severity			Mortality		
	Present (*n* = 51)	None (*n* = 20)	p value	Biological agent and/or MTX (*n* = 18)	Others (*n* = 53)	p value	Severe (*n* = 7)	Non-severe (*n* = 64)	p value	Non-survivors (*n* = 8)	Survivors (*n* = 63)	p value
*Streptococcus pneumoniae*	8 (15.7)^a^	5 (25.0)	0.496	1 (5.6)	12 (22.6)	0.161	4 (57.1)	9 (14.1)	0.018	3 (37.5)	10 (15.9)	0.156
Influenza virus	3 (5.9)	3 (15.0)	0.340	3 (16.7)	3 (5.7)	0.166	1 (14.3)	5 (7.8)	0.477	0 (0.0)	6 (9.5)	1.000
*Mycoplasma pneumoniae*	3 (5.9)	3 (15.0)	0.340	1 (5.6)	5 (9.4)	1.000	0 (0.0)	6 (9.4)	1.000	1 (12.5)	5 (7.9)	0.526
*Legionella* spp.	0 (0.0)	1 (5.0)	0.282	1 (5.6)	0 (0.0)	0.254	1 (14.3)	0 (0.0)	0.099	0 (0.0)	1 (1.6)	1.000
*Pseudomonas aeruginosa*	10 (19.6)	0 (0.0)	0.053	2 (11.1)	8 (15.1)	1.000	1 (14.3)	9 (14.1)	1.000	3 (37.5)	7 (11.1)	0.078
*Haemophilus influenza*	7 (13.7)	0 (0.0)	0.179	3 (16.7)	4 (7.5)	0.359	1 (14.3)	6 (9.4)	0.533	1 (12.5)	6 (9.5)	0.584
GNEB	4 (7.8)	0 (0.0)	0.571	2 (11.1)	2 (3.8)	0.265	0 (0.0)	4 (6.3)	1.000	0 (0.0)	4 (6.3)	1.000
*Chlamydophila pneumoniae*	1 (2.0)	1 (5.0)	0.487	1 (5.6)	1 (1.9)	0.445	1 (14.3)	1 (1.6)	0.189	0 (0.0)	2 (3.2)	1.000
*Moraxella catarrhalis*	5 (9.8)	0 (0.0)	0.312	1 (5.6)	4 (7.5)	1.000	2 (28.6)	3 (4.7)	0.073	1 (12.5)	4 (6.3)	0.460
*Stenotrophomonas maltophilia*	1 (2.0)	0 (0.0)	1.000	1 (5.6)	0 (0.0)	0.254	0 (0.0)	1 (1.6)	1.000	0 (0.0)	1 (1.6)	1.000
Others	4 (7.8)	1 (5.0)	1.000	1 (5.6)	4 (7.5)	1.000	1 (14.3)	4 (6.3)	0.414	0 (0.0)	5 (7.9)	1.000
Polymicrobial infection	14 (27.5)	3 (15.0)	0.471	4 (22.2)	13 (24.5)	0.505	3 (42.9)	14 (21.9)	1.000	2 (25.0)	15 (23.8)	0.677
Unknown	21 (41.2)	10 (50.0)	0.598	6 (33.3)	25 (47.2)	0.412	0 (0.0)	31 (48.4)	0.016	1 (12.5)	30 (47.6)	0.126

RA, rheumatoid arthritis; MTX, methotrexate; GNEB, Gram-negative enteric bacilli.

^a^Data are presented as means ± standard deviation or *n* (%).

### Severity on admission of pneumonia patients with and without RA

Seven (9.9%) RA patients and 218 (14.7%) non-RA patients met the criteria of severe pneumonia on hospital admission. The frequency of severe pneumonia in the RA patients did not differ from that in the non-RA patients (Table 1).

### Outcomes of pneumonia patients with and without RA

Eight (11.3%) RA patients and 74 (5.0%) non-RA patients died (Table 1). The mortality of the RA patients was statistically higher than that of non-RA patients (p = 0.050). All eight non-survivors with RA had respiratory comorbidities (Table 2).

### Risk factors for mortality from pneumonia

Multivariate analysis revealed older age, RA, severe pneumonia, and HCAP to be the independent risk factors for mortality from pneumonia (Table 5). The presence of respiratory comorbidities was found to be a significant risk factor for mortality in the univariate analysis, although this factor was not significant in the multivariate analysis.

**Table 5:** Multivariate analysis of the risk of mortality in the study patients.

Factor	n	Non-survivors	Univariate analysis			Multivariate analysis (final model)		
			OR	95% CI	p value^a^	OR	95% CI	p value
Age (≥65 years)	1009	72	4.073	(2.084, 7.958)	<0.001	2.233	(1.101, 4.526)	0.026
Male sex	1059	61	1.365	(0.821, 2.268)	0.230			
Smoker	947	47	0.846	(0.539, 1.327)	0.466			
Systemic disease	688	43	1.405	(0.900, 2.194)	0.134			
Pulmonary disease	823	58	2.218	(1.363, 3.608)	0.001			
Rheumatoid arthritis	71	8	2.409	(1.113, 5.213)	0.026	3.614	(1.552, 8.418)	0.003
Severity, severe	225	50	11.536	(7.203, 18.475)	<0.001	10.747	(6.584, 17.543)	<0.001
HCAP	507	47	2.940	(1.872, 4.617)	<0.001	2.372	(1.454, 3.867)	<0.001

OR, odds ratio; CI, confidence interval; HCAP, healthcare-associated pneumonia.

^a^Values represent p value for category against the reference.

## Discussion

We assessed the characteristics, severity, outcome, and etiology of pneumonia in RA patients. The characteristics of the RA patients with pneumonia differed from those of the non-RA patients with pneumonia in terms of certain patient demographics and in the microbial etiologies of pneumonia. Although the severity of the pneumonia in the RA patients did not differ significantly from that in the non-RA patients, mortality in the RA patients was significantly higher than that in the non-RA patients, and RA itself was an independent risk factor for mortality from pneumonia.

Vaccination against influenza and pneumococcus has been shown to reduce the prevalence of pneumonia in RA patients [8,9]. The present study showed that both *S*. *pneumoniae* and influenza virus were frequently isolated as pathogens of pneumonia in these patients. This result may be one of explanations for why vaccination against influenza and pneumococcus is associated with a lower prevalence of pneumonia in RA patients. Further prospective studies are needed to clarify the factors contributing to the reduction of the prevalence of pneumonia in RA patients.

A previous report indicated that structural diseases of the lung, smoking history, and cardiovascular diseases were the important risk factors of infections by *P*. *aeruginosa*, *H*. *influenzae*, and *M*. *catarrhalis* [6]. In the present study, infections by these three species were statistically more frequent in the RA patients than the non-RA patients, and all patients with these infections had respiratory comorbidities. The frequency of respiratory comorbidities in the RA patients was higher than that in the non-RA patients. Our data suggest that a higher frequency of respiratory comorbidities in RA patients may contribute to the difference in microbial etiologies from that in non-RA patients.

Reports in the literature even in the pre-steroid era suggest that patients with RA may have an increased susceptibility to infection [14]. RA patients have increased susceptibility to the development of pneumonia [3,5,15]. One possible explanation for this increased rate of pneumonia is the impaired immunologic abnormalities due to RA itself that involve the majority of circulating T cells, which develop from an early stage in the disease course [16]. RA therapy with corticosteroids and other immunomodulatory agents may also contribute to respiratory infection in patients complicated with RA. In fact, Widdifield *et al*. have suggested the associations of both previous and current anti-rheumatic drug use with increased risk of infection for both overall and specific infections [5]. In addition, the respiratory comorbidities that RA patients are often complicated with [15,17] should not be ignored. In the present study, 51 (71.8%) patients with RA had underlying respiratory diseases, which was statistically more frequent than that in the non-RA patients. The presence of these pulmonary and respiratory tract diseases may increase the risk of pneumonia [18].

Although the severity of pneumonia in the RA patients did not differ significantly from that in the non-RA patients, the mortality of the RA patients was significantly higher than that of the non-RA patients. We found that RA itself, older age (≥65 years), disease severity, and HCAP were identified as the independent risk factors for mortality.

Tsuchiya *et al*. investigated the outcome and causes of 71 deaths in 144 RA patients who had lung diseases directly associated with RA and reported that the cause of death in 9 (12.7%) of the 71 non-survivors was pneumonia [19], which suggests that pneumonia is an important cause of death in patients with underlying respiratory disease. Furthermore, disease-related factors such as immobility or the presence of extraarticular manifestations of RA were reported to contribute to the mortality of RA patients [20,21]. All 8 non-survivors in the present study had underlying respiratory diseases, and probably, their pulmonary function would also have been impaired. One reason for the difference in mortality between the RA and non-RA patients, and why RA itself was found to be an independent risk factor for mortality may be the impaired pulmonary function due to underlying respiratory diseases in the RA patients. The impaired pulmonary function, including the impaired local defense mechanism in patients with respiratory comorbidities or the impaired host defenses in RA patients, may contribute to its mortality. Physical activities such as performance status are frequently impaired in RA patients, which may have affected the present results [22]. It would be desirable to examine prognostic factors of pneumonia in RA patients in consideration of underlying disease severity in a future study.

The present study has several limitations. First, because it is a retrospective, observational, single-center study, the level of confidence is reduced, and the results may not be applicable in other settings. Second, we did not investigate the disease severity of RA, which may be an important risk factor for mortality. Third, we could not examine the influence of therapeutic drugs for RA on the outcome of pneumonia because few patients were given these drugs, especially MTX or biologics.

## Conclusion

Our results showed that there were several differences in patient demographics and etiologies between the RA patients and non-RA patients with pneumonia. The mortality from pneumonia in the RA patients was significantly higher than that in the non-RA patients, and RA itself was an independent risk factor for mortality from pneumonia. One possible explanation for the difference in clinical characteristics, etiology, and mortality between the RA patients and non-RA patients was the difference in the frequency of underlying respiratory disease. Careful attention should be paid to treating pneumonia in RA patients with respiratory comorbidities.
